# Neonatal mitochondrial abnormalities due to PINK1 deficiency: Proteomics reveals early changes relevant to Parkinson׳s disease

**DOI:** 10.1016/j.dib.2015.11.070

**Published:** 2015-12-17

**Authors:** Lance M. Villeneuve, Phillip R. Purnell, Kelly L. Stauch, Howard S. Fox

**Affiliations:** Department of Pharmacology and Experimental Neuroscience, University of Nebraska Medical Center, Omaha, NE 68198, United States

## Abstract

Parkinson׳s disease (PD), the second most common neurodegenerative disorder, affects roughly 7–10 million people worldwide. A wide array of research has suggested that PD has a mitochondrial component and that mitochondrial dysfunction occurs well in advance of the clinical manifestation of the disease. Previous work by our lab has categorized the mitochondrial disorder associated with Parkinson׳s disease in a PINK1 knockout rat model. This model develops Parkinson׳s disease in a spontaneous, predictable manner. Our findings demonstrated PINK1-deficient rats at 4 months of age had mitochondrial proteomic and functional abnormalities before the onset of Parkinsonian symptoms (6 months) such as the movement disorder, loss of midbrain dopaminergic neurons, or the progressive degeneration present at 9 months. With this in mind, our group investigated the PINK1 knockout genetic rat model at postnatal day 10 to determine if the observed alterations at 4 months were present at an earlier time point. Using a proteomic analysis of brain mitochondria, we identified significant mitochondrial proteomic alterations in the absence of mitochondrial functional changes suggesting the observed alterations are part of the mitochondrial pathways leading to PD. Specifically, we identified differentially expressed proteins in the PINK1 knockout rat involved in glycolysis, the tricarboxylic acid cycle, and fatty acid metabolism demonstrating abnormalities occur well in advance of the manifestation of clinical symptoms. Additionally, 13 of the differentially expressed proteins have been previously identified in older PINK1 knockout animals as differentially regulated suggesting these proteins may be viable markers of the PD pathology, and further, the abnormally regulated pathways could be targeted for therapeutic interventions. All raw data can be found in Supplementary Table 1.

## Specifications Table

**Table:** 

Subject area	Biology
More specific subject area	Neurobiology
Type of data	Protein expression table
How data was acquired	SWATH mass spectrometry (AB Sciex Triple-TOF 5600)
Data format	Normalized data
Experimental factors	Genetic ablation of the *Pink1* (A Parkinson׳s disease model)
Experimental features	Brains were isolated from post-natal day 10 (P10) males and age-matched controls (*n*=4). Mitochondria were isolated by a dual, sequential isolation and the resulting proteins were obtained for mass spectrometry.
Data source location	Omaha, NE
Data accessibility	The SWATH-MS data for the mitochondria isolated from the 10 day old PINK1 KO rat model can be accessed as Supplementary Table 1.

## Value of the data

• These methods demonstrate the ability to reproducibly quantify the brain mitochondrial proteome of animals at an early stage in life.
• These data provide differential expression changes in proteins known to directly influence mitochondrial function.
• These data suggest, when compared against previous studies, the fatty acid metabolic pathway is constitutively altered in a Parkinson׳s disease model and merits further investigation.

## 1 Data

Comparative analysis of the brain proteome of 10 day old PINK1 knockout animals revealed directed proteomic alterations present in the fatty acid metabolic pathway with significant expression deficits in 3-keto-CoA thiolase A (Acaa1a), 3-ketoacyl-CoA thiolase (mitochondrial) (Acaa2), and electron transfer flavoprotein subunit beta (Etfb). The rate-limiting enzyme of the citric acid cycle, isocitrate dehydrogenase (Idh1) was found to be significantly depressed and a key enzyme of glycolysis, fructose-bisphophate aldolase A (Aldoa) was decreased. When these data were compared against data obtained from older animals [1], similar expression profiles alterations were identified ( Table 1). In conjunction with other previously published data, these data suggest identify PINK1 pathways that are constitutively altered in Parkinson׳s disease.

## 2 Experimental design, materials and methods

### 2.1 Animals

All animal experiments were conducted with PINK1 KO and the Long Evans Hooded (LEH) control strains. PINK1 KO and LEH control female animals were housed together for 3 weeks to allow for synchronization of estrous cycle. Animals were then separated by genotype and bred. Animals for all experiments were born on the same day. All animals were 10 days old at the time of the experiments. Only male animals were used for the experiments. Gender was initially visually assessed but confirmed by necropsy. All protocols were conducted within NIH-approved guidelines with the approval and oversight of the University of Nebraska Medical Center IACUC.

### 2.2 Data-dependent analysis for building a library

A mitochondrial library was built as described previously [1], [2]. In short, mitochondrial lysates from B35, H19-7/IGF-IR, PC12 and RN33B cell lines were digested with trypsin, quantified, and fractionated by isoelectric focusing. Peptides from each fraction were prepared for mass spectrometry with Pierce C-18 PepClean Spin Columns (Thermo Fisher Scientific) in accordance with the manufacturer׳s instructions. Samples were dehydrated and resuspended in 6 μL of 0.1% formic acid for LC-MS/MS analysis. The mitochondrial isolation, protein and peptide processing was performed twice independently. The resulting 24 fractions of peptides were analyzed by nano-LC-MS/MS in SWATH-MS mode on the 5600 TripleTOF instrument. The SWATH-MS acquisition was performed using the published protocol [3]. The acquisition method was in data-dependent mode with one precursor scan followed by fragmentation of the 50 most abundant peaks. Precursor peaks with a minimum signal count of 100 were dynamically excluded after two selections for 6 s within a range ±25 mDa. Charge states other than 2–5 were rejected. Rolling collision energy was used. DDA files were searched in Protein Pilot. Combined results yielded a library of spectra representing 2004 proteins identified with high confidence (FDR≤5%).

### 2.3 Isolation of brain mitochondria for SWATH mass spectrometry and Seahorse analysis

Brains were rapidly isolated from 10 day old animals in both the PINK1 KO and LEH control groups. The brains were isolated from the animals, and the cerebellum was removed. After extraction, brains were immediately rinsed with ice-cold PBS to remove blood. The meninges were removed. Tissue was chopped and homogenized using a Dounce homogenizer. Brain mitochondria were isolated using differential centrifugation kit (Mitosciences, Eugene, OR) followed by an immunomagnetic purification using a kit with TOM-22 coupled to magnetic beads (MACS Miltenyi Biotec, Auburn, CA). Mitochondria for SWATH-MS were lysed in 4% sodium dodecyl sulfate (SDS) and protein concentration was quantified using a using a Pierce 660 assay with bovine serum albumin standards (Thermo Fisher Scientific, Rockford, IL).

### 2.4 Sample preparation for mass spectrometry and data-independent SWATH-MS analysis

Protein from the isolated mitochondria was digested with trypsin (Promega, Madison, WI) using the filter aided proteome preparation technique with a 20-μm filter (Pall Corporation, Ann Arbor, MI) as previously performed [2], [4], [5]. The resultant peptides were cleaned with an Oasis mixed-mode weak cation exchange cartridge (Waters, Milford, MA). Peptides were quantified using a Nanodrop (Thermo Fisher Scientific) in conjunction with Scopes method for protein quantitation [6].

### 2.5 Data-independent SWATH-MS analysis

Unfractionated samples of peptides from rat brain mitochondrial lysates were analyzed in quadruplicate (four biological replicates per age group) using SWATH data-independent analysis (DIA) as performed previously [5], [7]. All of the fragment ion chromatograms were extracted and automatically integrated with PeakView (v. 1.1.0.0). The raw peak areas as reported by PeakView were used for all the quantification calculations with no data processing (neither denoising nor smoothing) of any kind applied to the extracted ion chromatograms. To calibrate retention times, synthetic peptides (BiognoSYS; Zurich, Switzerland) were spiked-in the samples in accordance with the manufacturer׳s protocol. In accordance with previously published work [3], we selected 5 peptides and 5 transitions option for quantitative analysis and targeted data extraction for each peptide was performed. Samples were normalized to the area counts of the synthetic peptides. Briefly, for each peptide the fragment ion chromatograms were extracted using the SWATH isolation window set to a width of 10 min and 50 ppm accuracy for quantification purposes in accordance with previously established protocols [3].

### 2.6 Bioinformatic analysis

Significance was assessed using CyberT (http://cybert.ics.uci.edu/) using a sliding window of 101 and a Bayesian confidence coefficient of 12. Proteins were denoted as significantly altered if *p*<0.05 and the cumulative posterior probability of differential expression (Cum. PPDE) >0.95. A functional association protein network was generated using STRING (Version 10; http://string-db.org/) and uploaded into Cytoscape (Version 3.2.1) to create an interactive network [8]. Largely of 12 proteins differentially expressed a group of 3 were involved in fatty acid metabolism while another group was composed of histones interacting with the mitochondria. To assess the isolation of mitochondrial proteins, MitoMiner was utilized (http://mitominer.mrc-mbu.cam.ac.uk/release-3.1/begin.do) [9].

## Funding

Funding support was provided by a grant from the Michael J Fox Foundation (grant # 9524). Additional support was provided by NIH grants 5R01DA030962 and 5P30MH062261.

## Figures and Tables

**Table 1:** List of significantly altered proteins in 10 day old PINK1 KO rats compared with expression at 4 and 9 months of age. Proteins identified as significantly altered in PINK1 KO rats at 10 days of age were compared against data from 4 and 9 month old animals [1]. Only proteins listed as significantly different in 2 (10 day and 4 month or 9 month) analyses were included. For all proteins, a Bayesian analysis was performed with the Bayesian coefficient=12. Multiple testing corrections were applied in the form of cumulative posterior probability of differential expression (Cum. PPDE). Proteins were listed as significantly different if *p*<0.05 and Cum. PPDE≥0.95. Protein expression values listed are log_2_ (PINK1 KO/LEH). Unlisted values denote proteins without sufficient confidence for quantification. Values highlighted in green or red showed significantly decreased or increased expression in PINK1 KO rats, respectively.

Uniprot	Protein	Gene	10 day (Log2 PINK1 KO/LEH)	*p*-value	PPDE	Cortex						Striatum					
						4 month (Log2 PINK1 KO/LEH)	*p*-value	PPDE	9 month (Log2 PINK1 KO/LEH)	*p*-value	PPDE	4 month (Log2 PINK1 KO/LEH)	*p*-value	PPDE	9 month (Log2 PINK1 KO/LEH)	p-value	PPDE
P21775	3-ketoacyl-CoA thiolase A, peroxisomal	Acaa1a	−1.21	1.93E−06	1.00	−1.03	2.50E−04	0.98	−0.44	4.32E−01	0.00				−0.29	6.22E−01	0.41
P13437	3-ketoacyl-CoA thiolase, mitochondrial	Acaa2	−0.47	8.66E−05	0.99	−0.45	1.90E−04	0.99	−1.07	2.17E−02	0.00	−0.68	7.99E−04	0.95	−0.37	2.74E−01	0.59
Q68FU3	Electron transfer flavoprotein subunit beta	Etfb	−0.57	1.44E−04	0.99	−0.60	5.12E−05	0.99	−0.14	7.41E−01	0.00	−1.00	2.78E−06	1.00	−1.20	8.79E−03	0.89
P05065	Fructose-bisphosphate aldolase A	Aldoa	−0.88	6.08E−08	1.00	−1.20	5.86E−12	1.00	−0.84	1.18E−02	0.00	−1.38	5.55E−08	1.00	−0.98	1.57E−02	0.86
P08753	Guanine nucleotide-binding protein G(k) subunit alpha	Gnai3	0.82	1.15E−09	1.00	0.66	1.04E−04	0.99	0.11	6.57E−01	0.00	0.48	5.96E−02	0.68	0.84	5.55E−03	0.91
Q6P747	Heterochromatin protein 1-binding protein 3	Hp1bp3	−0.53	8.62E−04	0.95	−0.52	8.23E−04	0.96	−0.33	2.96E−01	0.00	−0.55	2.22E−02	0.78	0.70	1.01E−01	0.73
Q00715	Histone H2B type 1	N/A	−0.77	6.55E−08	1.00	−0.77	3.85E−08	1.00	−0.47	1.07E−01	0.00	−0.30	1.10E−01	0.60	1.54	7.85E−06	0.99
P62804	Histone H4	Hist1h4b	−0.43	4.86E−05	0.99	−0.43	3.78E−05	1.00	−0.28	2.09E−01	0.00	−0.44	1.18E−02	0.83	1.39	2.12E−08	1.00
Q00238	Intercellular adhesion molecule 1	Icam1	1.36	4.29E−04	0.97	1.37	8.47E−04	0.96									
P41562	Isocitrate dehydrogenase [NADP]cytoplasmic	Idh1	−0.48	1.48E−04	0.99	−0.53	2.36E−05	1.00	0.19	7.19E−01	0.00	−0.63	9.35E−02	0.62	0.62	2.93E−01	0.57
Q5M9G9	Protein TBRG4	Tbgr4	0.86	2.80E−04	0.98	0.85	3.09E−04	0.98				0.90	3.15E−02	0.75			
Q4FZT0	Stomatin-like protein 2, mitochondrial	Stoml2	2.14	3.25E−05	1.00	2.15	3.62E−05	1.00	0.01	9.76E−01	0.00	−0.60	6.80E−03	0.87	−0.58	1.98E−01	0.64
P69897	Tubulin beta-5 chain	Tubb5	−0.56	1.16E−06	1.00	−0.39	7.32E−06	1.00	0.03	8.83E−01	0.00	0.29	1.67E−01	0.55	0.41	2.02E−01	0.64

## Footnotes

Appendix A Supplementary data associated with this article can be found in the online version at doi:10.1016/j.dib.2015.11.070.
